# Circulating cell free DNA and citrullinated histone H3 as useful biomarkers of NETosis in endometrial cancer

**DOI:** 10.1186/s13046-022-02359-5

**Published:** 2022-04-21

**Authors:** Livia Ronchetti, Irene Terrenato, Margherita Ferretti, Giacomo Corrado, Frauke Goeman, Sara Donzelli, Chiara Mandoj, Roberta Merola, Ashanti Zampa, Mariantonia Carosi, Giovanni Blandino, Laura Conti, Anna Maria Lobascio, Marcello Iacobelli, Enrico Vizza, Giulia Piaggio, Aymone Gurtner

**Affiliations:** 1 SAFU Unit, IRCCS - Regina Elena National Cancer Institute, via Elio Chianesi 53, 00144 Rome, Italy; 2 Clinical Trial Center - Biostatistics & Bioinformatics, IRCCS - Regina Elena National Cancer Institute, Rome, Italy; 3 Department of Women and Children Health, Gynecologic Oncology Unit, Fondazione Policlinico Universitario A. Gemelli, IRCCS - Università Cattolica del Sacro Cuore, Roma, Italy; 4 Oncogenomics and Epigenetics Unit, IRCCS - Regina Elena National Cancer Institute, Rome, Italy; 5 Clinical Pathology Unit, IRCCS - Regina Elena National Cancer Institute, Rome, Italy; 6 Gynecologic Oncology Unit, IRCCS - Regina Elena National Cancer Institute, Rome, Italy; 7 Anatomy Pathology Unit, IRCCS - Regina Elena National Cancer Institute, Rome, Italy; 8 Institute of Translational Pharmacology (IFT), National Research Council (CNR), Rome, Italy

**Keywords:** NETs, NETosis, Circulating cell-free DNA, Citrullinated Histone H3, Neutrophil Elastase, Tumor induced systemic effects, DNA size distribution, Liquid biopsy, DNA cfDNA fragmentation pattern

## Abstract

**Background:**

Cancer mortality is mainly caused by organ failure and thrombotic events. It has been demonstrated that NETosis, a chromatin release mechanism implemented by neutrophils, may contribute to these lethal systemic effects. Our aim was to investigate NETosis biomarkers in endometrial cancer (EC).

**Methods:**

The experiments were conducted on 21 healthy subjects (HS) with no gynecological conditions, and on 63 EC patients. To assess the presence of NETosis features, IHC and IF was performed using antibodies against citrullinated histone H3 (citH3), neutrophil elastase (NE) and histone 2B. Serum levels of cell free DNA (cfDNA), cell free mitochondrial DNA (cfmtDNA) and citH3 were measured by qPCR using one microliter of deactivated serum, and by ELISA assay respectively. Fragmentation pattern of serum cfDNA was analyzed using the Agilent 2100 Bioanalyzer and High Sensitivity DNA Chips. Receiver operating characteristic (ROC) analysis was used to identify a cut off for cfDNA and cfmtDNA values able to discriminate between ECs and HSs. Correlation analysis and multiple correspondence analysis (MCA) between cfDNA, mtcfDNA, citH3 and blood parameters were used to identify the potential association among serum parameters in EC grades.

**Results:**

We demonstrated the presence of NETosis features in tissues from all EC grades. Serum cfDNA and cfmtDNA levels discriminate ECs from HSs and a direct correlation between citH3 and cfDNA content and an inverse correlation between cfmtDNA and citH3 in EC sera was observed, not detectable in HSs. MCA indicates cfDNA, cfmtDNA and citH3 as features associated to G1 and G2 grades. A correlation between increased levels of cfDNA, citH3 and inflammation features was found. Finally, serum nucleosomal cfDNA fragmentation pattern varies in EC sera and correlates with increased levels of cfDNA, citH3, lymphocytes and fibrinogen.

**Conclusion:**

Our data highlight the occurrence of NETosis in EC and indicate serum cfDNA and citH3 as noninvasive biomarkers of tumor-induced systemic effects in endometrial cancer.

**Supplementary Information:**

The online version contains supplementary material available at 10.1186/s13046-022-02359-5.

## Background

Tumor-induced systemic effects, such as thrombosis, organ failure and metastasis, are the cause of nearly all cancer-related mortalities. It has been demonstrated that the rapid DNA release mechanisms of activated immune-competent cells contribute to these systemic effects [1–4]. During immune responses, neutrophils are among the first cells to reach the site of inflammation. The increased number of tumor-associated neutrophils is linked to poor outcomes in different type of cancers, and many patients with advanced cancer show high levels of blood neutrophils [5–7]. It has been shown that various tumors are capable to predispose circulating neutrophils to produce neutrophil extracellular traps (NETs) causing systemic thrombosis and thromboembolism which are often associated with human cancers, through a biological process called NETosis [4, 8–14]. NETs are composed of nuclear DNA in a web-like structures extruded from neutrophils and mixed with granular and some cytoplasmic constituents, such as neutrophil elastase (NE), myeloperoxidase (MPO), and citrullinated histone H3 (citH3), in response to infections or cancer burden [15]. Recently, several studies have unveiled unexpected functions of NETosis in promoting tumor development including tumor growth, metastasis and angiogenesis and in protecting tumor cells from cytotoxic immune cells [12, 16–22]. This evidence suggests that NETosis may provide a novel tool, potentially targetable, for cancer treatment [14, 23–25]. Moreover, several studies indicate that the mechanism of NETosis can provide an explanation for the elevated circulating cell-free DNA (cfDNA) release in blood stream in pathologic conditions [26, 27] and strongly suggest that these NET-associated molecules are potential tumor biomarkers [5, 28–30].

We have recently demonstrated the potential of cfDNA as a simple and inexpensive biomarker in endometrial cancer (EC), particularly related to lymphovascular space invasion (LVSI) status [5, 28]. Despite EC generally has a good prognosis, about a quarter of patients present an unfavorable prognosis due to the advanced stage of disease at the time of diagnosis. Therefore, more efforts are needed to discover new biomarkers useful for early diagnosis and prognosis.

In the present study, a retrospective analysis was conducted on tissues and liquid biopsies to evaluate the presence of NETosis features in EC and their potential usefulness for diagnosis and/or prognosis. We investigated preoperative cfDNA quantitative and qualitative content, serum levels of citH3 and systemic inflammatory indicators such as fibrinogen, neutrophils, platelets and lymphocytes. We evaluated the prognostic significance of the combination of these factors in EC providing evidence on the possible involvement of NETosis formation in tumor-induced systemic effects.

## Methods

### Patient cohort

All healthy volunteers and EC patients were recruited at the Regina Elena National Cancer Institute. We collected serum samples from 21 healthy volunteers with no gynecological conditions, and 63 EC patients. Patients included in this study were not previously selected, but randomly chosen and recruited between 2014 and 2018. According with the histologic grade, we analyzed samples from 9 G1, 33 G2 and 21 G3 ECs. Supplementary table 1 depicts clinical-pathological characteristics of patients enrolled in this study.

### Immunohistochemistry

Citrullinated Histone H3 expression was evaluated by immunohistochemistry (IHC) on formalin-fixed paraffin-embedded (FFPE) tissues using rabbit polyclonal anti-Histone H3 (citrulline R2 + R8 + R17) (Abcam) at the dilution of 1:50 (citrate-based pH 6.0 epitope retrieval solution). Immunoreactions were revealed by a streptavidin–biotin enhanced immunoperoxidase technique in an automated stainer (Bond III, Leica Biosystem, Milan, Italy), according to the manufacturer’s instructions. The evaluation of histological slides was performed by the two observers (MC and LR) independently using two scoring systems that were subsequently compared. With the first evaluation, the percentage of stained cells out of total infiltrating cells was estimated. The sample was considered positive with a percentage of staining ≥ 15%. In the second evaluation, a standard point scale for staining intensity was used, with positive slides being scored 1 + , 2 + and 3 + using a positive control slide being taken as 3 + . Strong staining involves > 50% of the cells with score 2 + or 3 + . Moderate staining involves from 25 to 50% of the cells with score 1 + or 2 + . Weak staining involves < 25% of cells with score 1 + .

### Immunofluorescence

Immunofluorescence staining to observe the expression of NETosis markers was performed on formalin-fixed paraffin-embedded (FFPE) tissues utilizing the following primary antibodies: rabbit polyclonal anti-Elane (Atlas) at the dilution of 1:50 (citrate based pH 9.0 epitope retrieval solution); chicken polyclonal anti-Histone H2B (Abcam) at the dilution of 1:500 (citrate based pH 9.0 epitope retrieval solution) rabbit polyclonal anti-Histone H3 (citrulline R2 + R8 + R17) (Abcam) at the dilution of 1:50 (citrate based pH 6.0 epitope retrieval solution). The following were used as secondary antibodies: Cy^TM^2 Affinipure Donkey Anti-rabbit (Jackson Immuno Research) at the dilution of 1:400, CY^TM^3 Affinipure Donkey Anti-chicken (Jackson Immuno Research) at the dilution of 1:400. The colorations were read and interpreted by using a fluorescence confocal microscope. Immunoreactivity was evaluated by two investigators (LR and AG) and discordant cases were subsequently discussed and agreed upon. Thermo Scientific DAPI Nuclear Counterstains was used for nuclear staining.

### Blood processing

Venous blood of cancer patients were obtained before surgery and before the beginning of any treatment. The blood tests were obtained within 24 h for all patients as routine clinical practice before surgery. NLR, MLR and PLR were defined as the absolute neutrophil count in peripheral blood divided by the absolute lymphocyte count, the absolute count of monocyte divided by the absolute lymphocyte count, and the absolute platelet count in peripheral blood divided by the absolute lymphocyte count, respectively. For serum processing, blood samples were collected in Vacutainer tubes without anticoagulant and processed within 1–4 h. After collection the blood was allowed to clot at room temperature. The blood serum was separated by centrifugation at 1000–2000 × g for 10 min in a refrigerated centrifuge and stored at − 80 °C.

### Serum deactivation

Twenty μL of each serum sample were mixed with 20 μL of a preparation buffer (2.5% of tween 20, 50 mmol/L Tris–HCl, and 1 mmol/L EDTA). This mixture was digested with proteinase K (20 μg) solution for 50 min (Promega) at 56 °C, followed by 5 min of heat deactivation and insolubilization for 10 min at 95 °C. After subsequent centrifugation of 10,000 × g for 5 min, supernatant was used as a template for each quantitative real-time polymerase chain reaction (qRT-PCR) using SYBR Green Power Up Master Mix (Thermo Scientific) followed by evaluation of the average of CT values from triplicate reactions from Real Time PCR software.

### DNA extraction

Isolation from 1 ml of blood serum was performed with a commercial kit for blood DNA according to manufacturer’s specifications from Qiamp Circulating Nucleid Acid kit (QIAGEN). Concentration and purity of the extracted DNA were evaluated by performing a Qubit Fluorometric Quantification.

### Bioanalyzer analysis

DNA was isolated from 1 ml of blood serum with a commercial kit for blood DNA according to isolation manufacturer’s specifications from Qiamp Circulating Nucleid Acid kit (QIAGEN). Microfluidic electrophoresis using the Agilent 2100 Bioanalyzer and High Sensitivity DNA Chips (Agilent technologies Inc., Palo Alto, CA, USA) was performed to assess DNA fragment length for a size range between 35 and 10.380 base pairs (bp) based on manufacturer’s recommended protocol. Bioanalyzer 2100 Expert Software was used for the analysis of electrophoretic runs.

### Measurement of cfDNA levels

SYBR Gold Nucleic Acid Gel Stain (Invitrogen) was diluted first at 1:1000 in dimethyl sulphoxide (DMSO) and then at 1:8 in phosphate-buffered saline (PBS). Ten microliters of DNA solutions or sera were applied to a black 96-well plates. Forty microliters of diluted SYBR Gold were added to each well and fluorescence was measured with a 96well fluorometer at an emission wavelength of 535 nm and an excitation wavelength of 485 nm. Serum samples were diluted in PBS fivefold (20%). Assay was performed in triplicate. Standards were prepared with commercial Salmon sperm DNA.

### qPCR

One microliter of deactivated serum was used and both the mtDNA and nDNA content were measured by qPCR using SYBR Green Power Up Master Mix (Applied Biosystems, CA, USA) followed by evaluation of the average CT values from triplicate reactions from Real Time PCR software. Primers were designed and used for relative quantification for mtDNA to nDNA content. Two primer pairs were used for the amplification of two mitochondrial genes: MT-ND1 and mtDNA 16S. One primer set was used for the amplification of the single-copy nuclear gene 36B4. Primers sequences: forward ND1 5’-CCCTAAAACCCGCCACATCT-3’, reverse ND1- 5’-GAGCGATGGTGAGAGCTAAGGT-3’; forward mtDNA 16S 5’-CAGCCGCTATTAAAGGTTCG-3’, reverse: mtDNA 16S 5’-CCTGGATTACTCCGGTCTGA-3’, forward 36B4 5’-CAGCAAGTGGGAAGGTGTAATCC-3’, reverse: 36B4 5’-CCCATTCTATCATCAACGGGTACAA-3’. To determine the mtDNA content relative to nDNA, the following equations were used: ΔCT = (mtDNA CT- nDNA CT). Relative mitochondrial DNA content = 2-^ΔCT^.

### ELISA assay

To quantify CitH3 in the blood, we employed the citrullinated Histone H3 (Clone 11D3) ELISA Kit (Cayman) following the manufacturer’s recommended protocol.

### Statistics

The examined variables were not normally distributed, as verified by the Shapiro–Wilk test, thus the most suitable non-parametric test was applied to perform comparisons between groups. Receiver operating characteristic (ROC) curves and the relative area under the curve (AUC) were calculated for all continuous variables of interest. Youden’s index was used to maximize the difference between sensitivity and specificity in order to individuate the optimal cut-off for each parameter. Multiple correspondence analysis (MCA), a descriptive/exploratory technique designed to analyze simple two-way and multi-way table, was used to obtain an overview of the potential association among parameters. *P*-values < 0.05 were considered statistically significant. The Spearman’s Rho coefficient was applied to conduct correlation analysis. The SPSS (version 21.0) was used for all statistical analyses.

## Results

### NETosis occurs in all grades of EC

**Fig. 1:**
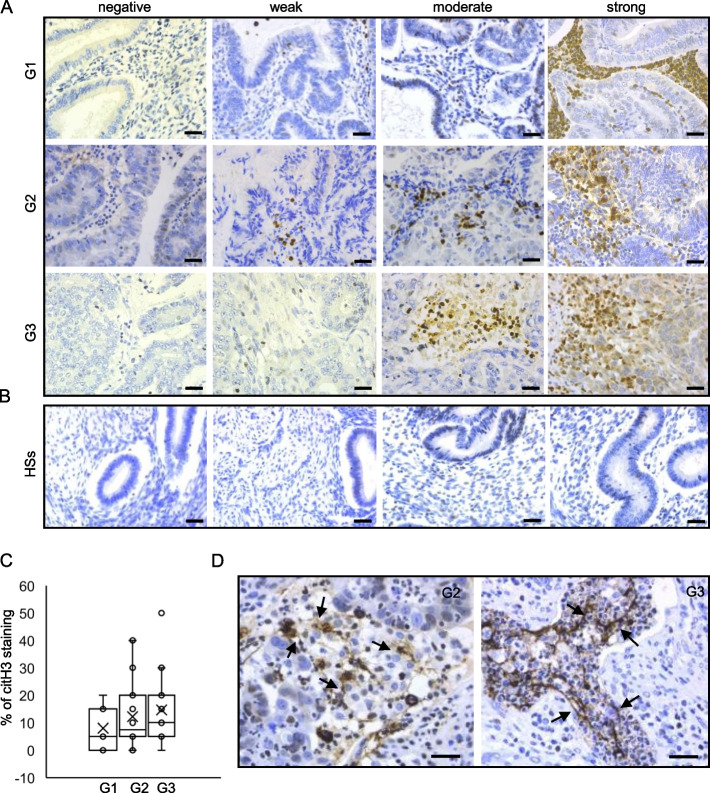
citH3 staining is present in all endometrial tumor grades. IHC staining of endometrial FFPE tissues with anti-citH3 antibody observed by light microscopy, scale 30 um, 20 × objective. **A** Staining of citH3 in the leukocyte infiltrate in all EC grades (G1, G2, G3), ranging from low to high signal intensity. **B** Healthy subjects (HSs) are negative for the staining. **C** Box-plots of citH3 staining % in leucocytes infiltrate in 48 EC samples. Boxes extend from the 25th to 75th percentiles, the horizontal line in the box represent median. X indicates mean value. **D** Images of G2 and G3 EC tissues (40 × objective). Black arrows indicate citH3 release into the extracellular space. All scale bars indicate 30 mm

To start to assess the presence of NETosis features in EC, we performed IHC using an antibody against citH3 on EC tissues and, as control, healthy subject (HS) endometrial tissues. The disease characteristics of the enrolled patients are described in Supplementary Table 1. Representative IHC staining are shown in Fig. 1A. In all EC grades, the analysis of citH3 staining revealed a variable level of staining ranging from negative to strong positive samples (Fig. 1A). Although the presence of this variability, of note, among all samples, the majority of tumors (81.25%) presented an infiltrate stained for citH3, with a high IHC score (+ 2, + 3) in 60% of G1, 78,6% of G2, and 93,4% of G3 (Fig. 1C). By contrast, the 4 HSs were negative for this staining (Fig. 1B). Interestingly, the percentage of leucocytes expressing citH3 increases from G1 to G3 (Fig. 1C). Notably, we could detect citH3 positive staining in the nucleus and cytoplasm of neutrophils as well as extracellular filaments resembling NETs (Fig. 1D).

**Fig. 2:**
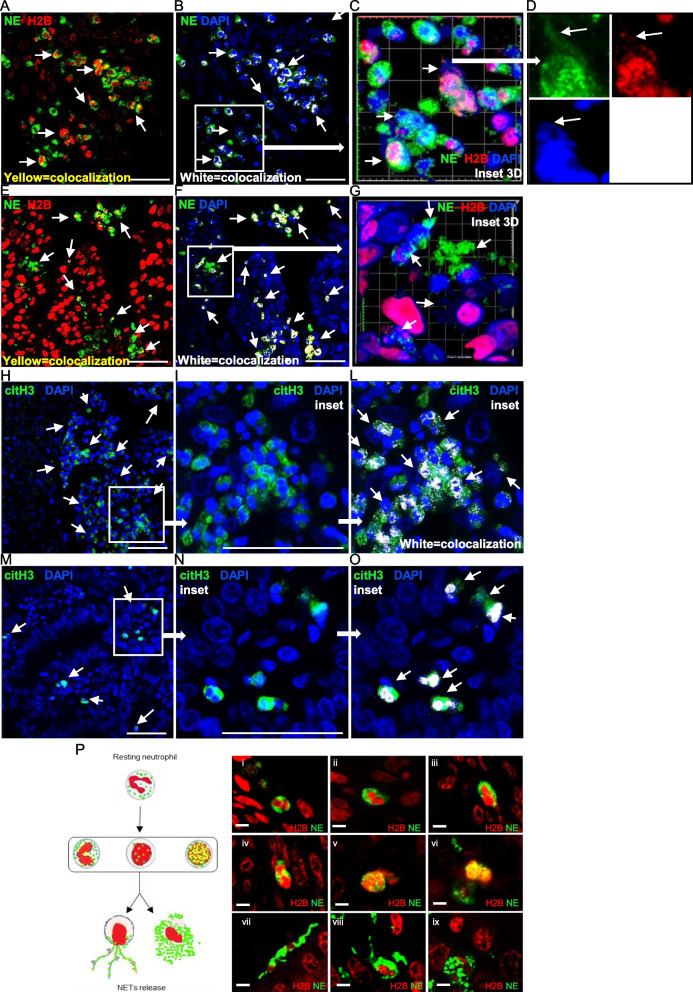
Cosnfocal microscope imaging of NETosis in EC specimens. **A**, **E** Two representative samples (grade G2 and G3) stained with anti-NE and anti-histone H2B antibodies. The yellow color on the images indicates the colocalization between the two markers. **B**, **F** Stainings of the same samples as A and E with anti-NE antibody and DAPI. The white color on the images indicates the colocalization between NE and DNA; when in the nucleus, colocalization strongly suggests neutrophils activation. 40 × oil objective, confocal single stack. **C**, **G** 3D reconstruction of magnifications of the areas inside the white squares in B and F respectively. Merge of 3 fluorochromes are shown. **D** Single staining of panel C detail highlights extracellular extrusion of DNA and citH3. 60 × oil objective. **H**, **M** Stainings of the same samples as A and E respectively with anti-citH3 antibody and DAPI. 40 × oil objective, confocal single stack. **I**, **L**, **N**, **O** Magnifications of the areas inside white squares in H and M respectively. In L and O the white color indicates colocalization between citH3 and DNA in the nucleus, cytoplasm and extracellular space. 60 × oil objective. Thin white arrows indicate the areas of colocalization and extracellular extrusion of NE, DNA and citH3. All scale bars indicate 50 µm. **P** Schematic representation (left) flanked by IF images taken from EC tissues (right), of the various stages of NETosis. Panels I, II, III show resting neutrophils, with trilobed nucleus and intact cytoplasm containing granules of NE. In IV, V, VI the nucleus has lost its classic trilobed shape, a yellow color is observed due to the nuclear co-localization of the two markers, testifying the transfer of NE into the nucleus. Panels VII, VIII and IX show the release of neutrophil extracellular traps (NETs). All scale bars indicate 5 µm

To further understand whether the citH3 positivity accounts for NETosis by activated neutrophils, we performed confocal immunofluorescence experiments on 30 EC tissues that allowed us to visualize of both intra- and extracellular localization of NE, histone H2B, DNA and citH3. Representative images of 2 EC tissues (G2 and G3 respectively), show a colocalization of NE and H2B (Fig. 2A, 2E; colocalization in yellow). On the same tissue sections, a colocalization of NE and DAPI in the nucleus and cytoplasm of tumor-infiltrating neutrophils has also been observed (Fig. 2B, 2F; colocalization in white). 3D image magnifications of triple staining (NE, H2B, DAPI) on representative selected areas (withe boxes) further highlight the colocalization among the three markers. Of note, extrusions of DNA (blue) and NE (green) from tumor-infiltrating neutrophils are also visible (white arrows) and are associated with the loss of the trilobate nuclear shape (Fig. 2C, D, G). citH3 staining on EC tissues from the same patients indicates strong neutrophil activation (Fig. 2H, 2M). Magnification of a representative field highlights colocalization (in white) between citH3 and DNA both in the nucleus, in the cytoplasm and extracellular space (Fig. 2I, L, N, O). Tissues Images of staining of single antibodies are shown in Supplementary Fig. 1.

Of note, we identified the different stages of NETosis process. We were able to visualize resting neutrophils (Fig. 2P, panels I, II, III), early stages of the process characterized by cytoplasmic to nucleus NE translocation (Fig. 2P panels IV, V, VI), and late stages featured by loss of nuclear lobulation, membrane rupture and nuclear chromatin and NE release, (Fig. 2P, panels VII, VIII, IX).

In conclusion, combined IHC and IF analysis demonstrate the presence of NETosis processes in all grade of EC tissues.

### Serum citH3 levels correlate with increased cfDNA content in EC

**Fig. 3:**
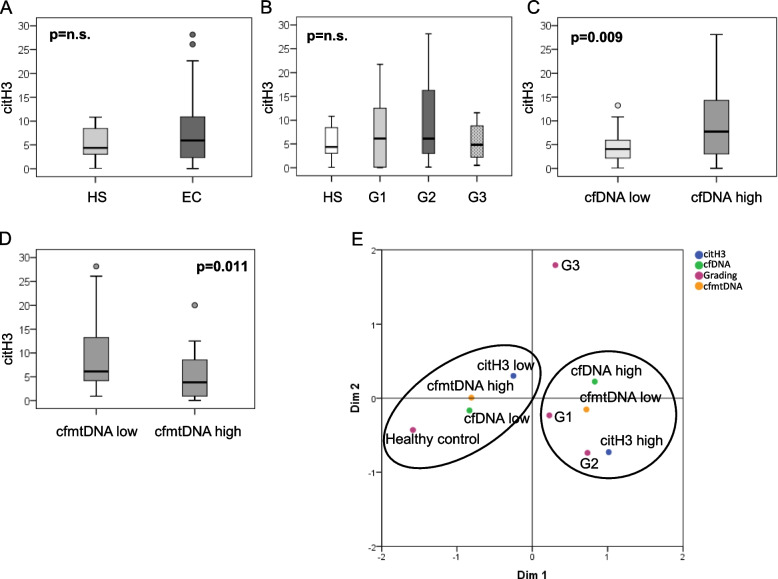
citH3 levels are directly related to cfDNA levels and inversely related to cfmtDNA in EC sera. Box-plots of citH3 content in (**A**) HS and EC sera (**B**) G1, G2, and G3 EC grades. **C** Cluster analysis of citH3 levels in EC sera displaying a cfDNA content ≤ 712,3 or > 712, (**D**) cfmtDNA content ≤ 16,5 or > 16,5. Boxes extend from the 25th to 75th percentiles, the horizontal line in the box represent median. *P* values, Mann–Whitney test in panel A-C-D; Kruskall-Wallis test in B; n.s.: not significant. **E** Identification of EC-DNA-characteristics related to different grade. Plot of the two-dimension multiple corresponding analysis (MCA) among HSs and EC grades (G1, G2, G3) of cfDNA, cfmtDNA and citH3 categorized according to cut-offs individuated by ROC analysis

Serum citH3 levels have been previously demonstrated to be a biomarker of NETosis as well as cfDNA [26, 27]. We have previously demonstrated an increase of cfDNA and a decrease of cfmtDNA in EC sera compared with sera of HSs [5]. Based on this, we thought to investigate the relationship between serum citH3 levels and cfDNA (both total and mitochondrial) in 63 EC and 22 HS sera. According to our previously results, we observed a significant increase of cfDNA and a decrease of cfmtDNA contents in EC samples compared with HSs (Supplementary Fig. 2A, B). In relation to EC grades the two features significantly discriminate G2 and G3 grades from HSs (Supplementary Fig. 2C, D). By receiver operating characteristic (ROC) analysis, we identified a cut-off for cfDNA and cfmtDNA values with high predictive ability, being able to strongly discriminate between ECs and HSs (Table 1). Median values of citH3 were, instead, comparable in EC and HS sera (Fig. 3A) as well as in different EC grades (Fig. 3B). Interestingly, categorizing by ROC cut off cfDNA and cfmtDNA value distributions, we observed that citH3 levels were significantly higher in samples with high values of cfDNA (Fig. 3C) and low values of cfmtDNA (Fig. 3D). Next, we investigated the correlation between these three features. Results showed a significant, although fair, direct correlation between citH3 and cfDNA as well as an inverse correlation with cfmtDNA (Table 2). No correlation has been found in HSs (data not shown). Analyzing the correlations among histological grades, we found a significant direct correlation between citH3 and cfDNA in G2 samples. Instead, an inverse correlation between citH3 and cfmtDNA, as well as between cfDNA and cfmtDNA, occurs in G1 samples (Table 3). In agreement with these results, MCA analysis locates HSs and ECs in two distinct groups (Fig. 3E). High levels of citH3 and cfDNA and low levels of cfmtDNA are located closed to G1 and G2. On the contrary, HS samples are located close to citH3 low, cfDNA low, and cfmtDNA high levels. G3, in this analysis, is not associated with any of these features (Fig. 3E). To understand whether our results are specific to histone citrullination, we analyzed the levels of another post-translational histone modification, the histone H3 di-methylation in lysine 9 (H3k9me2). First, we observed that the serum levels of the two modified histones, citH3 and H3k9me2, were inversely correlated in a highly significant manner in EC samples (Table 4). Second, when we compared the two modified histones in relation to cfDNA and mtDNA levels, we observed that they behave with an inverse trend. Indeed, H3k9me2 levels decrease in EC sera with high cfDNA content (Table 4). Third, categorizing by ROC cut off cfDNA value distributions, we observed that H3k9me2 levels were lower in samples with high values of cfDNA (Supplementary Fig. 3). No correlation has been found in HSs (data not shown). These results demonstrated that the two post-translational histone modifications are mutually exclusive and, more importantly, di-methylation in lysine-9 is more present in EC samples with less cfDNA levels.

**Table 1:** ROC analysis. (Status = Healthy subjects vs endometrial cancer patients)

	**Area Under the Curve (AUC) (95%CI)**	***p*-value**	**Test direction**	**Sensitivity**	**Specificity**	**Cut-off**
cfDNA	0.838 (0.76–0.92)	** < 0.001**	Higher values indicate Cancer	0.67	1.00	**712.3**
cfmtDNA	0.854 (0.77–0.94)	** < 0.001**	Lower values indicate Cancer	0.65	0.95	**16.5**
citH3	0.583 (0.44–0.73)	0.339	Higher values indicate Cancer	0.33	0.93	9.2

**Table 2:** Correlation analysis between cfDNA, cfmtDNA, citH3, blood parameters in EC sera. Spearman’s Rho correlation coefficient

	**cfmtDNA**	**citH3**	**Neu**	**Neu/Lym**	**Mono/Lym**	**Mono**	**Lym**	**Platelets**	**Fibr**
**cfDNA**	-0,083 N = 52	0,439_**_ *N* = 54	0,250	-0,014	-0,049	0,360_**_	0,415_**_	0,318*	0,498_**_
			*N* = 52	*N* = 52	*N* = 52	*N* = 52	*N* = 52	*N* = 52	*N* = 52
**cfmtDNA**	1,000	-0,384_**_	-0,111	-0,104	-0,008	0,085	0,088	0,223	0,028
		*N* = 49	*N* = 47	*N* = 47	*N* = 47	*N* = 47	*N* = 47	*N* = 47	*N* = 47
**citH3**		1,000	0,184	-0,051	-0,094	0,213	0,297^*^	0,244	0,225
			*N* = 47	*N* = 47	*N* = 47	*N* = 47	*N* = 47	*N* = 47	*N* = 42

^*^p < 0.005; **p < 0.001

*Neu* Neutrophilis, *Mono* Monocites, *Lym* Lymphocites, *Fibr* Fibrinogen

**Table 3:** Correlation analysis between cfDNA, cfmtDNA, citH3, and blood parameters stratified by EC grade. Spearman’s Rho correlation coefficient

Grade 1	cfmtDNA	citH3	Neu/Lym	Mono/Lym	Fibr	Platelets	Neu	Lym	Mono
**cfDNA**	-0,683_*_	0,483	0,429	0,500	0,857_*_	0,143	0,786_*_	0,464	0,750
	*N* = 9	*N* = 9	*N* = 7	*N* = 7	*N* = 7	*N* = 7	*N* = 7	*N* = 7	*N* = 7
**cfmtDNA**	1,000	-0,733_*_	-0,071	0,143	-0,357	0,143	-0,429	-0,536	-0,107
		*N* = 9	*N* = 7	*N* = 7	*N* = 7	*N* = 7	*N* = 7	*N* = 7	N = 7
**citH3**		1,000	-0,071	-0,393	0,000	0,321	0,429	0,679	0,143
			*N* = 7	*N* = 7	*N* = 7	*N* = 7	*N* = 7	*N* = 7	*N* = 7
**Grade 2**	**cfmtDNA**	**citH3**	**Neu/Lym**	**Mono/Lym**	**Fibr**	**Platelets**	**Neu**	**Lym**	**Mono**
**cfDNA**	-0,087	0,415_*_	-0,091	-0,144	0,529_**_	0,370	0,262	0,453_*_	0,347
	*N* = 27	*N* = 28	*N* = 28	*N* = 28	*N* = 28	*N* = 28	*N* = 28	*N* = 28	*N* = 28
**cfmtDNA**	1,000	-0,350	0,219	0,132	0,260	0,106	0,069	-0,042	-0,073
		*N* = 25	*N* = 25	*N* = 25	*N* = 25	*N* = 25	*N* = 25	*N* = 25	*N* = 25
**citH3**		1,000	-0,194	-0,029	0,276	0,258	0,045	00,271	0,249
			*N* = 24	*N* = 24	*N* = 24	*N* = 24	*N* = 24	*N* = 24	*N* = 24
**Grade 3**	**cfmtDNA**	**citH3**	**Neu/Lym**	**Mono/Lym**	**Fibr**	**Platelets**	**Neu**	**Lym**	**Mono**
**cfDNA**	0,388	0,333	-0,135	-0,167	0,379	0,434	0,069	0,346	0,254
	*N* = 16	*N* = 17	*N* = 17	*N* = 17	*N* = 17	*N* = 17	*N* = 17	*N* = 17	*N* = 17
**cfmtDNA**	1,000	-0.286	-0,386	-0,271	-0,054	0,420	0,289	0,101	0,301
		*N* = 15	*N* = 15	*N* = 15	*N* = 15	*N* = 15	*N* = 15	*N* = 15	*N* = 15
**citH3**		1,000	0,071	-0,141	0,131	0,226	0,141	0,074	0,053
			*N* = 16	*N* = 16	*N* = 16	*N* = 16	*N* = 16	*N* = 16	*N* = 16

^*^p < 0.005; **p < 0.001

*Neu* Neutrophilis, *Mono* Monocites, *Lym* Lymphocites, *Fibr* Fibrinogen

**Table 4:** Correlation analysis between cfDNA, cfmtDNA, citH3, H3k9me2 in EC sera. Spearman’s Rho correlation coefficient

	**cfDNA**	**cfmtDNA**	**citH3**
**H3k9m3**	**-0,293**	0,335	-0,750_**_
	*N* = 27	*N* = 25	*N* = 27
**cfDNA**	1,000	-0,083	0,439_**_
	*N* = 63	*N* = 52	*N* = 54
**cfmtDNA**	-0,083	1,000	-0,384_**_
	*N* = 52	*N* = 52	*N* = 49
**citH3**	0,439_**_	-0,384_**_	1,000
	*N* = 54	*N* = 49	*N* = 54

^*^p < 0.005; **p < 0.001

In conclusion our data indicate that elevated serum cfDNA concentration is associated with citH3, a well-known biomarker of NETosis, in G1 and G2 EC patients, supporting the idea that they could be useful to monitor NETosis process by non-invasive liquid biopsies thus opening the way for new therapeutic strategies in EC.

### Fragmentation pattern of cfDNA in ECs is associated with citH3 serum levels

cfDNA often appears as a ladder of fragments of different sizes. Interestingly, some experimental evidence suggests that the nature of the fragments changes in cancer patients having prognostic potential [31–33]. We have previously demonstrated that cfDNA integrity index, determined by the ratio of qPCR Alu247/qPCR-Alu115, provides prognostic value in EC [28].

Several studies have highlighted that the sensitivity of cfDNA to DNAses is strictly dependent on nucleosome positioning, a mechanism where there are also involved histone post-translational modifications, on epigenetic signature and genetic structure [34–37]. Consequently, cfDNA sizing analysis has emerged as a new strategy to increase the cfDNA diagnostic power. However, it is not known yet which is the fragmentation pattern of released cfDNA derived from NETosis. Based on these considerations, we wondered whether citH3 levels correlate with a specific cfDNA fragment size distribution. To address this issue, we analyzed purified double strand cfDNA samples of 44 ECs and 14 HSs (Supplementary Table 2), with Agilent DNA High Sensitivity Kit. This kit is developed for sizing and quantifying DNA fragments from 35 to 10.380 bp. We found that all HSs, with only one exception, have a cfDNA fragmentation profile with a nucleosomal DNA ladder pattern corresponding to mono- (195 bp), di- (390 bp), tri- (585) and poly- (from 585 to 9050 bp) nucleosomes (Supplementary Fig. 4). Unlike HSs, EC samples present more variable patterns. All EC samples, regardless of grade, exhibit the short peak fragment of 195 bp usually associated with apoptotic events [31–33]. Among these, 41% of samples present only this short peak (cf mono nucleosome pattern, cfMNP), while 59% of them also have an accumulation a poly-nucleosomes from 1000 to 9050 bp (cf total nucleosome patterns, cfTNP). Moreover, while the intensity of the HS peaks never exceeds 50 fluorescence units (FU), 30% of ECs present peaks above 50 FU reaching, in some cases, 500 FU (Supplementary Fig. 4). This result is in agreement with increased cfDNA levels observed in ECs (Supplementary Fig. 2A).

**Fig. 4:**
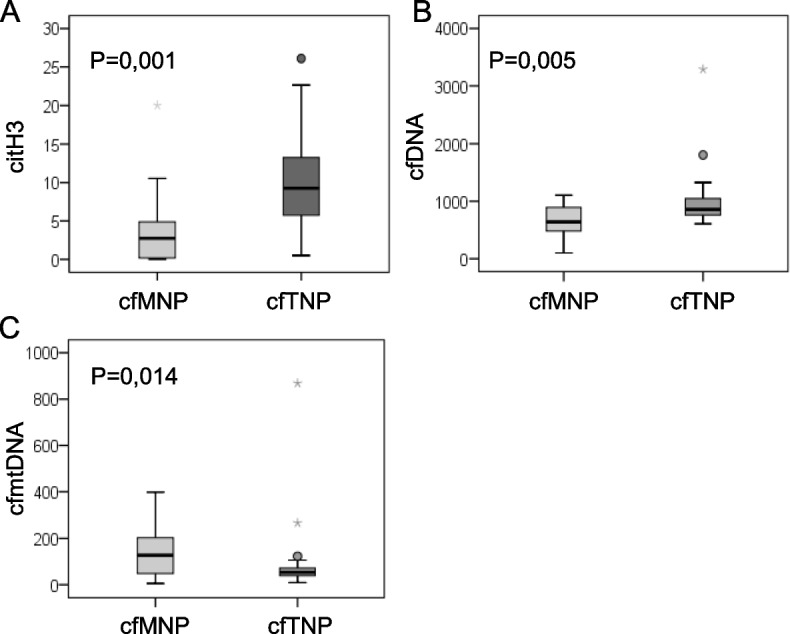
A higher citH3 content is associated with the presence of cfTNP in EC sera. Box plots showing citH3 (**A**), cfDNA (**B**), cfmtDNA (**C**) levels in the two subgroups of EC serum samples that present cfMNP or cfTNP respectively. The upper border of the box indicates the upper quartile (75th percentile) while the lower border indicates the lower quartile (25th percentile), and the horizontal line in the box the median. *P* values (Mann–Whitney non-parametric test), n: 19 cfMNP and 25 cfTNP

To understand whether a fraction of cfDNA is derived from NETosis, we clustered EC samples into two groups, depending on the presence of cfTNP or only cfMNP and we investigated the association of each group with serum citH3, cfDNA and cfmtDNA levels. This analysis demonstrated that the presence of cfTNP was associated with higher concentrations of citH3 and cfDNA and with a lower cfmtDNA content (Fig. 4A, B, C).

**Fig. 5:**
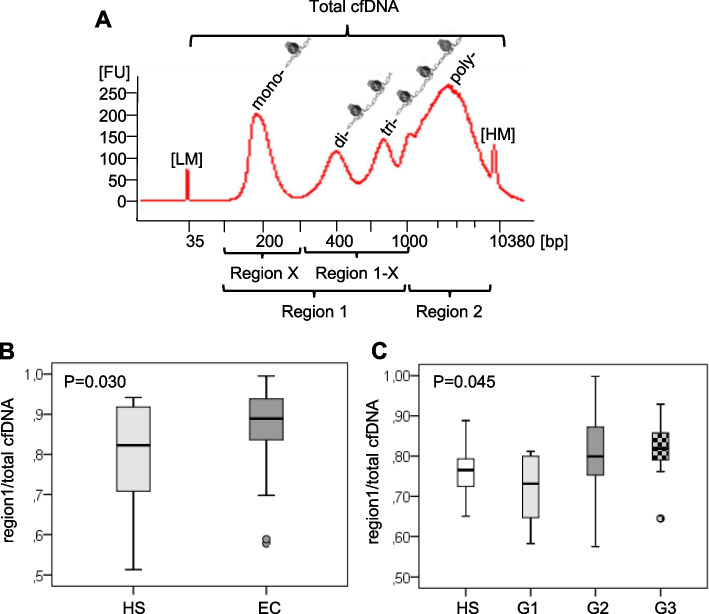
Amount of mono- di- and tri- nucleosome cfDNA related to the total DNA increases in EC serum samples. **A** Schematically representation of the different DNA regions analyzed (region X, 1, 1-x, 2, total). **B** Box-plots of cDNA region 1 DNA content in HSs and ECs. **C** Box-plots of cDNA region 1 DNA content in HSs and in different EC grades (G1, G2, and G3). FU: fluorescence units (proportional to the DNA molarity/concentration), LM: low marker, HM: high marker. Boxes extend from the 25th to 75th percentiles, and the horizontal line in the box the median

Then to investigate whether a specific cfDNA region accounts for these associations, we split total cfDNA in four regions encompassing different DNA lengths (Fig. 5A). We found significant direct correlations between all regions (spanning from mono- to poly-nuclosomes) and citH3 with significant values in G1 and G2 (Table 5). This result is cancer-specific since any correlation has been found in HSs (data not shown). Conversely, no correlation or very weak correlation between citH3 and mono- and di-nucleosomal regions occurs in G3 (Table 5). These results are in agreement with the correlation found between cfDNA and citH3 levels only in lower grades (Table 2, 3), and with MCA results (Fig. 3E) that associate the three parameters citH3, cfDNA, and cfmtDNA in G1 and G2 but not in G3. Consequently, the three parameters cfDNA levels, cfDNA ladder, and citH3 levels can distinguish low from high grade ECs.

**Table 5:** Correlation among citH3 levels and concentration of region 1, region 2, region X, region 1-X and total cfDNA, in EC stratified by grading. Spearman’s Rho correlation coefficient

		**Region 1**	**Region 2**	**Region X**	**Region 1-X**	**Total cfDNA**
G1 (*N* = 8)	**citH3**	0,905^**^	0,714^*^	0,929**	0,929**	0,905^**^
G2 (*N* = 21)	**citH3**	0,781_**_	0,684_**_	0,708**	0,734**	0,742_**_
G3 (*N* = 13)	**citH3**	0,637_*_	0,434	0,549	0,687*	0,549

^*^p < 0.005; **p < 0.001;

All together these findings suggest that the poly-nucleosomal DNA ladder pattern, spanning from 195 to 10.000 bp, may reflect NETosis in G1 and G2 EC samples. Moreover, the presence of NET-related longer cfDNA fragments suggests an impairment on NETosis clearance in these samples. Finally, from a quantitative point of view, only region 1 is significantly greater in ECs than in HSs respect to the total cfDNA, with an increasing trend from G1 to G3 grades (Fig. 5B, C).

### Correlation between inflammatory/coagulation features and NETosis

**Fig. 6:**
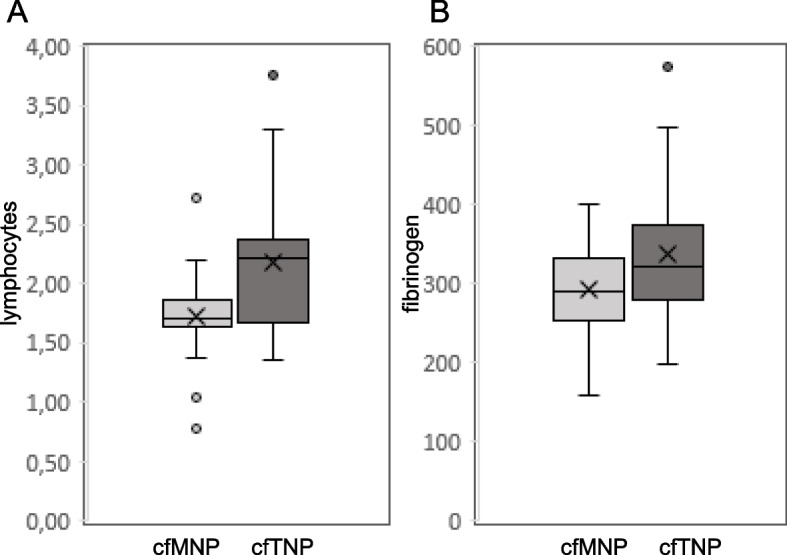
Inflammatory markers increase in the subgroup of cfTNP EC samples. Box plot of lymphocyte (**A**), and fibrinogen (**B**) levels in the two subgroups of EC serum samples that present cfMNP or cfTNP cfDNA ladder pattern. Boxes extend from the 25th to 75th percentiles, and the horizontal line in the box the median. n: 19 cfMNP and 25 cfTNP. X indicates mean value

To assess the possible network between inflammatory features and NETosis, we measured the relationship between inflammatory cell counts and citH3, cfDNA and cfmtDNA levels. A positive correlation was found between citH3 levels and lymphocyte counts (Table 2) in ECs while cfDNA content positively correlates with monocyte, lymphocyte, platelet counts and fibrinogen levels (Table 2). Next, stratification by EC grades disclosed a consistent and positive relationship of cfDNA with both neutrophils and fibrinogen in G1, and with lymphocytes and fibrinogen in G2 (Table 3). No correlation was found between NET markers and inflammatory features in G3 (Table 3). To evaluate whether these markers are associated with the poly-nucleosomal DNA ladder pattern, we measured levels of the inflammatory markers clustering in the two EC subgroup, cfMNP and cfTNP. The results show an increasing trend of lymphocyte counts and fibrinogen levels in cfTNP (Fig. 6).

In conclusion our data suggest that citH3 NETosis biomarker, cfDNA content and its fragmentation pattern may reflect the lymphocyte-mediated inflammatory response and coagulation status in ECs discriminating G3 from G1 and G2 grades.

## Conclusions

In the present study, a retrospective analysis was conducted on tissues and liquid biopsies to evaluate the presence of NETosis features in endometrial cancer and their possible usefulness for diagnosis and/or prognosis. We investigated preoperative cfDNA quantitative and qualitative content, serum levels of citH3 and systemic inflammatory indicators such as fibrinogen, neutrophils, platelets and lymphocytes. We evaluated the prognostic significance of the combination of these factors providing evidence on the possible involvement of NETosis formation in tumor-induced systemic effects.

First, by IF and IHC analysis we found that the majority of tumor tissue samples present an infiltrate of leucocytes positive for the NETosis biomarker citH3, with an increasing trend from G1 to G3 grade. To our knowledge, this is the first time that NET structures are described both intracellularly in infiltrate cells and in the extracellular space in endometrial cancer specimens. Unexpectedly, serum citH3 levels do not increase in cancer grades and, consequently, we do not observe an association of citH3 tissue staining with its serum levels. This may be due to different mechanisms of cfDNA clearance in tissues versus sera, and/or to the different timing of collection of tissues and blood samples.

Next, we found that serum cfDNA and cfmtDNA levels are useful to discriminate between endometrial cancer patients and healthy subjects. Interestingly, citH3 levels are significantly higher in sera of endometrial cancer samples presenting cfDNA and cfmtDNA levels higher and lower respectively, to the ROC cut off. Further, MCA analyses discriminate EC patients from healthy subjects, locating G1 and G2 grades far from G3 and close to high level of citH3, cfDNA and low levels of cfmtDNA. In agreement, we highlight positive and inverse correlations between cfDNA and cfmtDNA respectively, and citH3 levels in G1 and G2. These results strongly support the idea that cfDNA and cfmtDNA levels are the results, at least in part, of NETosis process. Furthermore, as neutrophils are characterized by containing a very limited number of mitochondria compared with other leukocytes [38], the decrease in serum cfmtDNA content supports the scenario of NETosis activation in endometrial cancer tissues. Noteworthy, corroborating the specificity of our data is the evidence that levels of H3k9me2 are inversely correlated with increased concentrations of citH3 and cfDNA in EC patients. These new results are in agreement with previous findings describing NETosis as a process of chromatin decondensation. Indeed, it has been demonstrated that upon neutrophils stimulation, the activated peptidylarginine deiminase PAD4 translocates into the nucleus and catalyzes the conversion of positively-charged arginine (including Arg 8) into neutral citrulline present on H3, resulting in the inhibition of chromatin ionic interactions. This event leads to a decrease in lysine 9 methylation of histone H3, a marker of closed chromatin, and prevents heterochromatin protein 1 (HP1) from binding to chromatin thus promoting its decondensation [39, 40]. Further studies should be done to better characterize the epigenetic landscapes of cfDNA in EC sera in order to find new possible cfDNA-based biomarkers.

In recent years, serum analysis of cfDNA in cancer patients has attracted the attention of researchers and clinicians in numerous fields and has opened promising perspectives for diagnosis and prognosis [31–33]. The cfDNA sizing analysis in cancer is an issue of great actuality and little is known about the dimension of cfDNA fragments released by neutrophils during NETosis. It is well known that the DNA ladder and nucleases involved change in different extracellular DNA release processes such as apoptosis and necrosis, and this is reflected in various DNA fragmentation profiles [41]. Indeed, fragments of 160/195 bp are a hallmark of apoptosis. Differently necrosis mainly generates fragments > 10 kb. Less is known about the DNA fragmentation during NETosis, however evidence underlined that long DNA fragments are released from neutrophils and that the enzymes involved in their clearance are principally DNAse1L3 and DNAseIII [42]. The sensitivity of cfDNA to DNAses is strictly dependent on its nucleosomal, epigenetic signature reflecting also the genetic peculiarities of the tissue of origin [34–37]. Consequently, cfDNA sizing analysis has emerged as a new strategy to increase the cfDNA diagnostic power. Therefore, in the last years several strategies have been used to study cfDNA size profile such as qPCR, next generation sequencing and microfluidic electrophoresis. In this regard, we have recently demonstrated that evaluation of cfDNA integrity index, calculated as qPCR-Alu247 value /qPCR-Alu115, may represent a complementary tool to help EC stratification by demonstrating that cfDNA integrity was significantly higher in advanced EC grade [28]. In addition, we have found an increased neutrophil-to-lymphocyte ratio and its positive correlation with cfDNA levels in sera of EC patients compared with sera of healthy subjects [5]. These data suggested that longer cfDNA fragments, not attributable to apoptosis, might derive from an active release from leukocytes during the NETosis process, thereby contributing to the serum pool of cfDNA and specific histone modifications such as citH3. Here, we decipher the cfDNA fragmentation profile in endometrial cancer patient sera and its relationship with NETosis by microfluidic electrophoresis experiments that allowed us to analyze DNA regions ranging from 1000 to 10,000 bp. Indeed, our results highlight the presence of cfDNA fragments spanning from mono- to poly-nuclosomes, all of which could, in G1 and G2 grades, derive from NETosis process, given the consistent correlation of these fragments with high serum levels of citH3. To our knowledge this is one of the few studies investigating serum cfDNA ladder upper to 1000 bps and its correlation with NETosis in an oncological disease.

A growing number of studies report that citH3, a marker of NETosis, increases in sera of different cancer types and predicts poor prognosis [12]. Moreover, other markers of NETosis, such as MPO and NE, have already been associated with tumor progression in colorectal cancer, breast cancer, gastric adenocarcinoma [29, 30, 43]. A novelty of our study resides in the analysis of the three combined parameters citH3, cfDNA, and cfmtDNA in EC serum samples. Indeed, collectively our data demonstrate that serum cfDNA and cfmtDNA are novel non-invasive biomarkers of NETosis in endometrial cancer and pave the way for their diagnostic and prognostic use in combination with serum citH3 content. Further studies should be done to investigate the prognostic value of our three combined biomarkers also in other tumor types.

### Supplementary Information

**Additional file 1:** **Supplementary figure 1.** Confocal microscope imaging of IF single stainings in EC specimens. **Supplementary figure 2. **cfDNA levels increase in EC samples and are inversely related to cfmtDNA. **Supplementary figure 3.** H3k9me2 levels are inversely related to cfDNA levels in EC sera. **Supplementary figure 4.** DNA fragmentation pattern analysis of cfDNAin HS and EC serum samples.

**Additional file 2:** **Supplementary Table 1.** Patient’s characteristics. **Supplementary table 2.** Schematic representation of samples used in the different experimental strategies.

## Data Availability

The datasets used and/or analyzed during the current study are available from the corresponding author on reasonable request.

## Abbreviations

cfDNA: Cell free DNA
cfmtDNA: Cell free mitochondrial DNA
cfMNP: Cf Mono nucleosome pattern
cfTNP: Cf Total nucleosome patterns
citH3: Citrullinated histone H3
EC: Endometrial cancer
FU: Fluorescence units
FFPE: Formalin-fixed paraffin-embedded
HSs: Healthy subjects
MPO: Myeloperoxidase
MCA: Multiple correspondence analysis
NETs: Neutrophil extracellular traps
NE: Neutrophil elastase
ROC: Receiver operating characteristic
