# Correction: Circulating cell free DNA and citrullinated histone H3 as useful biomarkers of NETosis in endometrial cancer

**DOI:** 10.1186/s13046-023-02826-7

**Published:** 2023-10-24

**Authors:** Livia Ronchetti, Irene Terrenato, Margherita Ferretti, Giacomo Corrado, Frauke Goeman, Sara Donzelli, Chiara Mandoj, Roberta Merola, Ashanti Zampa, Mariantonia Carosi, Giovanni Blandino, Laura Conti, Anna Maria Lobascio, Marcello Iacobelli, Enrico Vizza, Giulia Piaggio, Aymone Gurtner

**Affiliations:** 1 SAFU Unit, IRCCS - Regina Elena National Cancer Institute, via Elio Chianesi 53, Rome, 00144 Italy; 2 Clinical Trial Center - Biostatistics & Bioinformatics, IRCCS - Regina Elena National Cancer Institute, Rome, Italy; 3 Department of Women and Children Health, Gynecologic Oncology Unit, Fondazione Policlinico Universitario A. Gemelli, IRCCS - Università Cattolica del Sacro Cuore, Roma, Italy; 4 Oncogenomics and Epigenetics Unit, IRCCS - Regina Elena National Cancer Institute, Rome, Italy; 5 Clinical Pathology Unit, IRCCS - Regina Elena National Cancer Institute, Rome, Italy; 6 Gynecologic Oncology Unit, IRCCS – Regina Elena National Cancer Institute, Rome, Italy; 7 Anatomy Pathology Unit, IRCCS - Regina Elena National Cancer Institute, Rome, Italy; 8 Institute of Translational Pharmacology (IFT), National Research Council (CNR), Rome, Italy

**Correction:** ***J Exp Clin Cancer Res*** **41, 151 (2022)**

10.1186/s13046-022-02359-5

Following publication of the original article [1], errors were identified in the Methods and Declarations section. The updated texts re given below and the changes have been highlighted in **bold typeface**.

Methods

Patient cohort

Patients included in this study were not previously selected, but randomly chosen and recruited between 2014 and **2018**.

Declarations

Ethics approval and consent to participate

**Signed informed consents were obtained from all patients and healthy volunteers. Twenty-one healthy volunteers have been enrolled on the basis of an experimental protocol approved by the Ethical Committee of the Regina Elena National Cancer Institute (Rome, Italy, ifo_058, IFO_AOO.REGISTRO UFFICIALLE.U.0013925.14-12-2017. Amended on 27-07-2023). Sixty-three patients enrolled between 2014 and 2018 have signed an institutional biobank informed consent. Information about patients was obtained by reviewing their medical charts.**

The corrections do not affect the overall result or conclusion of the article. The original article has been corrected.
