# Supplementary material for: Circulating cell free DNA and citrullinated histone H3 as useful biomarkers of NETosis in endometrial cancer
Source: J Exp Clin Cancer Res. 2022 Apr 21;41:151. doi: 10.1186/s13046-022-02359-5 (PMC9027343; doi:10.1186/s13046-022-02359-5)
Supplement: Supplementary file 1 — Additional file 1: Supplementary figure 1. Confocal microscope imaging of IF single stainings in EC specimens. Supplementary figure 2. cfDNA levels increase in EC samples and are inversely related to cfmtDNA. Supplementary figure 3. H3k9me2 levels are inversely related to cfDNA levels in EC sera. Supplementary figure 4. DNA fragmentation pattern analysis of cfDNAin HS and EC serum samples. [file 13046_2022_2359_MOESM1_ESM.pdf]

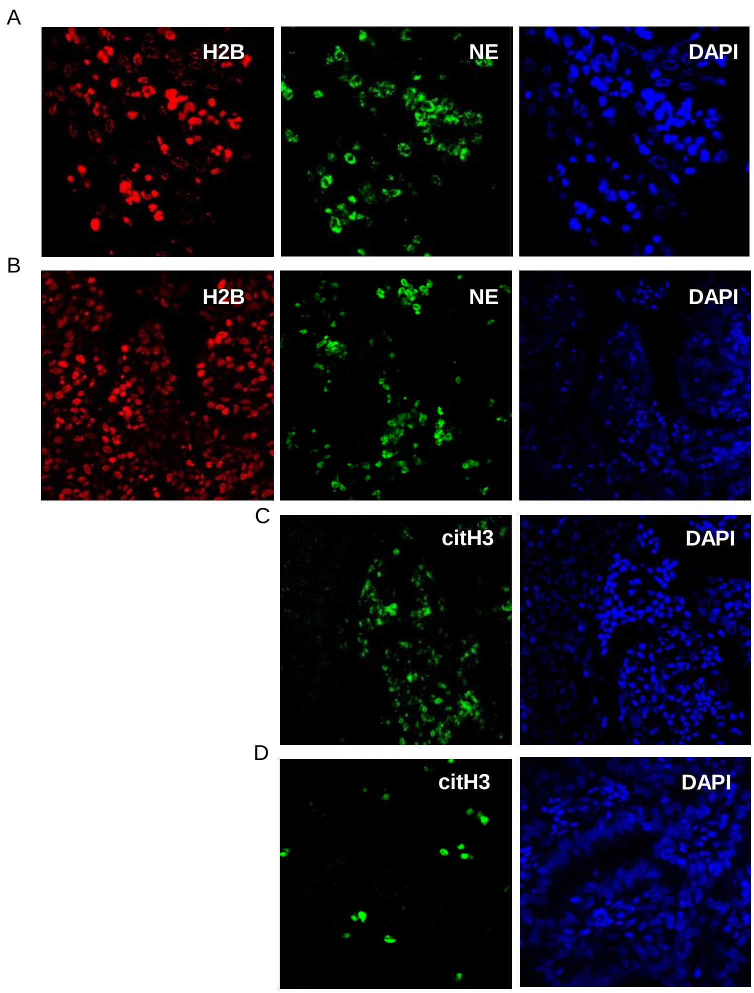

Supplementary figure 1. Confocal microscope imaging of IF single stainings in EC specimens.

IF single staining of EC tissues of the two representative patients of figure 1, with anti-histone H2B, anti-NE, DAPI, anti-citH3 antibodies. (A) Single staining of figure 1 panel A; (B) single staining of figure 1 panel E; (C) single staining of figure 1 panel H; (D) Single stain of figure 1 panel M. 40x oil objective, confocal single stack.

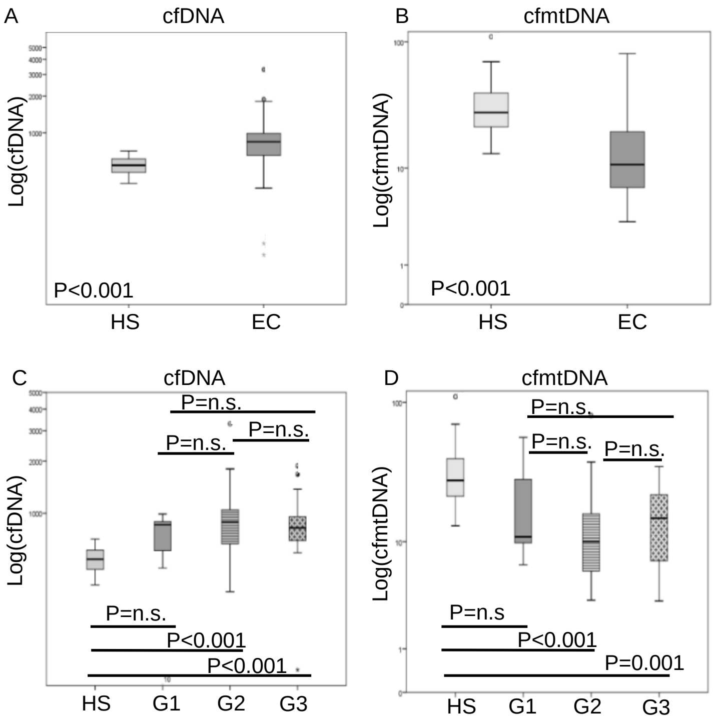

New supplementary figure 2: cfDNA levels increase in EC samples and are inversely related to cfmtDNA.

Box-plots of cfDNA levels (A and C), cfmtDNA content (B and D) in HSs, ECs and G1, G2, and G3 of EC serum samples. Boxes extend from the 25th to 75th percentiles, the horizontal line in the box represent median. *P* values, Mann–Whitney test in A, B; Kruskall-Wallis test in C, D. n.s.: not significant.

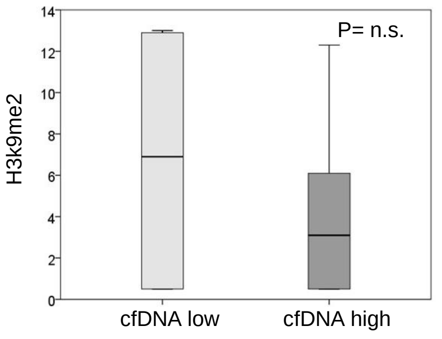

New Supplementary figure 3: H3k9me2 levels are inversely related to cfDNA levels in EC sera.
Cluster analysis of citH3 levels in EC sera displaying a cfDNA content ≤ 712,3 or >712.

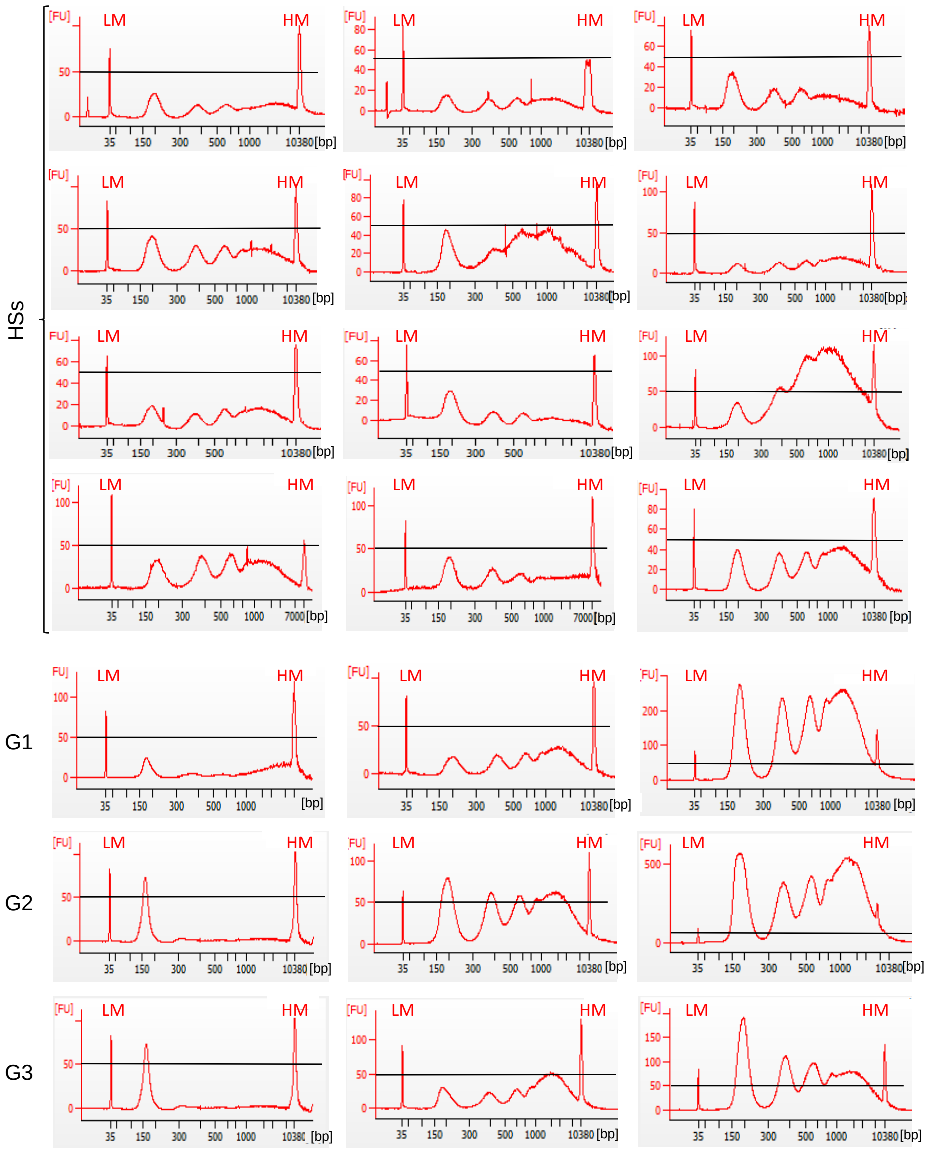

Supplementary figure 4: DNA fragmentation pattern analysis of cfDNA in HS and EC serum samples.

Representative electropherograms of our cohort of cfDNA samples (A) from HSs, (B) from different EC grades (G1, G2, and G3). FU: fluorescence units (proportional to the DNA molarity/concentration), LM: low marker, HM: high marker. Black lines define 50 FU.
