# Supplementary material for: Circulating cell free DNA and citrullinated histone H3 as useful biomarkers of NETosis in endometrial cancer
Source: J Exp Clin Cancer Res. 2022 Apr 21;41:151. doi: 10.1186/s13046-022-02359-5 (PMC9027343; doi:10.1186/s13046-022-02359-5)
Supplement: Supplementary file 2 — Additional file 2: Supplementary Table 1. Patient’s characteristics. Supplementary table 2. Schematic representation of samples used in the different experimental strategies. [file 13046_2022_2359_MOESM2_ESM.docx]

**Supplementary Table 1. Patient’s characteristics**

| **Parameters** | **Grading** | | | **p-value** |
| --- | --- | --- | --- | --- |
|  | **G1** | **G2** | **G3** |  |
|  | **N° (%)** | **N° (%)** | **N° (%)** |  |
| **N° cases** (63 total) | 9 (10) | 33 (39) | 21 (25) |  |
| **Age** |  |  |  | **0.046*** |
| Median (min-max) | 54 (48-66) | 60 (30-78) | 64 (44-80) |  |
| **FIGO stage** (63 total) |  |  |  | **0.005**** |
| IA | 9 (100) | 19 (58) | 4 (19) |  |
| IB | 0 | 10 (30) | 5 (24) |  |
| II | 0 | 3 (9) | 4 (19) |  |
| IIIA | 0 | 0 | 1 (3) |  |
| IIIB | 0 | 1 (3) | 1 (3) |  |
| IIIC1 | 0 | 0 | 2 (9) |  |
| IIIC2 | 0 | 0 | 4 (19) |  |
| **BMI** (63 total) |  |  |  | **0.638**** |
| <30 | 7 (78) | 21 (64) | 13 (62) |  |
| ≥30 | 2 (22) | 12 (36) | 8 (38) |  |
| **LVSI** (38 total) |  |  |  | **0.003**** |
| Present | 0 | 3 (14) | 10 (71) |  |
| Absent | 3 (100) | 14 (67) | 2 (14.5) |  |
| Not clear | 0 | 4 (19) | 2 (14.5) |  |
| **MI** (50 total) |  |  |  | **0.005**** |
| <50% | 7 (100) | 16 (57) | 4 (27) |  |
| >50% | 0 | 12 (43) | 11 (73) |  |

*Kruskall-Wallis non parametric test; ** qui-square test. BMI: Body mass index,

LVSI: Lymphovascular space invasion, MI: myometrial invasion

**Supplementary table 2. Schematic representation of samples used in the different experimental strategies.**

| **Experiment type** | **HS**  **N° of case** | **EC**  **N° of case** | **G1**  **N° of case**  **(% of total EC)** | **G2**  **N° of case**  **(% of total EC)** | **G3**  **N° of case**  **(% of total EC)** |
| --- | --- | --- | --- | --- | --- |
| **IHC/IF** | 4 | 48/28 | 7 (15)/4 (14) | 26 (54)/14 (50) | 15 (31)/10 (36) |
| **Serum citH3** | 14 | 54 | 9 (17) | 28 (52) | 17 (31) |
| **cfDNA** | 21 | 63 | 9 (14) | 33 (53) | 21 (33) |
| **cfmtDNA** | 20 | 55 | 10 (18) | 29 (53) | 16 (29) |
| **DNA size distribution analysis** | 14 | 44 | 10 (23) | 22 (50) | 12 (27) |
